# Making Sense of the Unique Pain of Survivors: A Psychoeducational Approach for Suicide Bereavement

**DOI:** 10.3389/fpsyg.2020.01244

**Published:** 2020-06-30

**Authors:** Isabella Berardelli, Denise Erbuto, Elena Rogante, Salvatore Sarubbi, David Lester, Maurizio Pompili

**Affiliations:** ^1^Department of Neurosciences, Mental Health and Sensory Organs, Faculty of Medicine and Psychology, Suicide Prevention Centre, Sant’Andrea Hospital, Sapienza University of Rome, Rome, Italy; ^2^Department of Psychology, Faculty of Medicine and Psychology, Sapienza University of Rome, Rome, Italy; ^3^Department of Psychology, Stockton University, Galloway, NJ, United States

**Keywords:** survivors, psychoeducation, groups, suicide, bereavement

## Abstract

Grief, guilt, abandonment, anger, shame, and rejection are the most common feelings experienced by suicide survivors, who differ from other bereaved individuals for the intensity of these feelings. Moreover, suicide risk and psychiatric disorders associated with suicidality are more frequent in people who have lost a loved person by suicide. Given the complexity and the consequences linked to the suicide of a loved person, it is necessary to act promptly. Among the various strategies, psychoeducation has proved effective for several mental disorders and for suicide bereavement. It is a therapeutic intervention aimed at identifying and understanding the psychological features associated with the mental pain of suicide survivors, to facilitate the management of the illness and the recognition of relationships in the social environment. We developed a psychoeducational group that took place at the Suicide Prevention Center of the Sant’Andrea Hospital in Rome. It was a homogeneous, finite-group composed of 8–12 suicide survivors and conducted by two trained psychologists supervised weekly by the Director of the Suicide Prevention Center. The intervention comprised 21 weekly sessions of 90 min. Each session concerned a determined topic and began with the presentation of the issue, continued with specific exercises, and finished with a group discussion. The main goals of the group were to provide support, normalize the reactions of the survivors, and assist them in reducing their emotional suffering and their thoughts about suicide, investigate the potential presence of suicide risk, implement prevention strategies, and integrate the loss of the loved person. The psychoeducational approach we delivered for suicide survivors allows individuals to interact with other individuals in the same situation in order to help them resume the normal course of life, placing the suicide of a loved person in a broader perspective.

## Introduction

With the purpose of developing a postvention group approach for suicide survivors, a new approach was developed and used at the Suicide Prevention Center in Rome. Dedicated researchers and clinicians in the area of suicidology provided a psychoeducational approach for managing the grief after the suicide of a loved one. Grief and other psychological, physical, and behavioral responses are the most frequent emotional reactions to suicide (Shear and Shair, 2005). The grief experienced after a suicide death, including the course and the duration, is similar to the grief process after other causes of death, but suicide survivors, may experience more shock or trauma due to the sudden and violent nature of the suicide (Cerel et al., 2009; Berman, 2011; Maple et al., 2017). Suicide survivorship is generally associated with traumatic experiences. Jordan and McIntosh (2011) reported that the “trauma of losing a loved one to suicide [w]as ‘catastrophic’—on a par with that of a concentration camp experience” (Jackson, 2003). While literature routinely reports that survivors experience trauma and are exposed to an excess of mental disorders, complicated grief, and suicide risk, there are, of course, people who resiliently overcome such a “traumatic event.” Comprehensive accounts on postvention are available and poignantly described the phenomenology of survivors of suicide (Andriessen et al., 2019). However, scholars have focused on the impact of suicide deaths on loved ones with relatively little attention on the diathetic aspects of their mental wellness prior to such experience. It is also essential to foster protective factors among such individuals, making sense of their resources, of how they coped with past traumatic experiences, and trying to implement tactics aimed at cultivating well-being and early detection of mental disorders. Grief experiences in suicide survivors are characterized by deep feelings of guilt, rejection, abandonment, anger, and shame about the death, in addition to the fear of being partly responsible for the suicide (Jordan et al., 1993; Cerel et al., 2013). Recently, Kõlves et al. (2019), comparing those bereaved by suicide and non-suicidal violent death, found significantly higher levels of psychological suffering including rejection, stigmatization, shame, and fear of being responsible in the group of suicide survivors 2 years after their loss (Kõlves et al., 2019). Moreover, the grief experience also depends on the relationship between the suicide and the suicide survivor and on the meaning that the survivor gives to the suicidal act (Tal et al., 2017). Jordan (2001, p. 91) suggested that the psychological pain of suicide survivors, “differs in three significant ways: the thematic content of grief, the social processes surrounding the survivor, and the impact that suicide has on family systems,” compared to that from other causes of death. Furthermore, the authors suggested that suicide bereavement contains prominent and intense “thematic issues,” including “(1) survivors feel that they caused the death directly, through mistreatment or abandonment, (2) they blame themselves for not anticipating the suicide, and (3) they frequently present feelings of rejection, abandonment, and anger” (Barrett and Scott, 1990; Cleiren and Diekstra, 1995). Several authors have stressed the association between the difficulty in understanding the suicide and several symptoms of complicated grief in suicide survivors (Currier et al., 2006). Survivors experience higher levels of rejection, shame, stigma, and blame than other bereaved people, although they share with them complicated grief, depression, hopelessness, PTSD symptoms, anxiety, and suicidal behaviors (Saarinen et al., 1999; Bellini et al., 2018). The psychological impact of suicide for both adults and adolescents seems to depend, in addition to the possible presence of psychiatric symptoms, on other factors including kinship, gender, and features related to the relationship with the suicide person (Andriessen et al., 2016).

There is also an increase in physical illness among suicide survivors, possibly due to the adoption of self-harming lifestyles, including the use of alcohol and unprescribed drugs (Erlangsen et al., 2017; Eng et al., 2019). Given the considerable emotional suffering of suicide survivors, suicidal ideation (SI) has received considerable clinical attention, especially in recent years (Sveen and Walby, 2008; Pompili et al., 2013). Several authors have reported a strong association between complicated grief and SI, with suicide survivors having a significantly higher risk of suicide spectrum disorders compared with people bereaved by other traumatic deaths (Mitchell et al., 2004; Pitman et al., 2014). Bereavement following a suicide is difficult to understand and, often, suicide survivors have difficulties in adapting psychologically to this event, becoming more vulnerable to physical complaints as well as psychiatric symptoms and suicide risk (Ellenbogen and Gratton, 2001; Jordan, 2001; Sveen and Walby, 2008). Suicide survivors present an increased suicide risk-related both to interpersonal difficulties and disruption of attachments and to their vulnerability to psychiatric disorders (Brown, 1998).

Regarding the stigma perceived by suicide survivors, studies have demonstrated that suicide survivors can be subjected to prejudice and discrimination, factors that may make their psychological suffering and their social isolation worse (Sudak et al., 2008; Scocco et al., 2017).

Much of the psychological pain perceived by suicide survivors results from the need to understand the motivations that led to suicide (Kaslow et al., 2011). Furthermore, suicide survivors often overestimate their role in contributing to the suicide death, not considering other features involved in suicide (Robins, 1981; Jordan, 2008). Survivors often experience emotional confusion and ambiguity, resulting, at least in part, from not understanding that suicide is a choice derived from intense psychological pain of the deceased in relation to, not only to mental illness, but also to relational and personal difficulties. In both cases, these questions are a source of intense suffering for survivors, especially when suicide is conceptualized as a voluntary act (Jordan, 2008).

Given the complexity and the ambiguity of the emotional reactions, feelings, and symptoms to which suicide survivors are exposed, it is essential to implement a series of interventions aimed, not only at understanding and assessing the psychological state of survivors, but also attending to the psychological needs of people in this condition (Cerel et al., 2019). Several non-pharmacological techniques have been used as a treatment for this population, and, among these, psychoeducation seems to have good clinical efficacy. In this article, we summarize a postvention psychoeducational technique for suicide survivors, showing in detail the psychoeducational model that is used in our Suicide Prevention Center.

### Psychoeducational as a Postvention Intervention for Suicide Survivors

Shneidman (1969) defined the term postvention as support for suicide survivors. Postvention includes a package of interventions with the aim of facilitating recovery after the suicide of a loved person as well as assessing and preventing psychological symptoms and distress in suicide survivors (Andriessen, 2014). The World Health Organization [WHO] (2014) recognized suicide survivors’ support as an important strategy in suicide prevention programs, and postvention approaches are used in many countries. A recent study indicated that, although the number of psychological postvention approaches for people bereaved by suicide is limited, the results suggest good clinical efficacy (Andriessen et al., 2019).

Among the various postvention strategies currently available, psychoeducation has shown effectiveness in preventing relapses for several mental disorders and can be considered to be a valid option also for postvention for suicide survivors. Psychoeducation is an intervention dedicated to the recognition of relationships in the social environment, and the psychoeducational model follows research on emotion expression (EE). This type of intervention identifies some psychological factors, including empathy, hyper-involvement, and emotional hostility, recurrent in some families (Brown et al., 1962; Leff et al., 1982). In the 1980s, “psychoeducational interventions” were developed for reducing the high levels of EE in the families of psychiatric patients (Vaughn and Leff, 1976; Falloon et al., 1982).

The word “psychoeducation” was first used by Anderson et al. (1980) for describing a non-pharmacological approach that included four basics elements. These included teaching the patients about their illness, and working on problem-solving, communication, and self-assertion techniques (Anderson et al., 1980). Over time, these elements have been enriched and merged, creating the most recent models of psychoeducation (Mottaghipour and Tabatabaee, 2019). Several studies have demonstrated the effectiveness of psychoeducational family interventions as compared with standard treatments for psychiatric disorders (Miklowitz et al., 2000; Colom et al., 2009). Psychoeducational programs have demonstrated good effectiveness in reducing the impact of several psychiatric symptoms in schizophrenia, bipolar disorder, and major depressive disorder, improving pharmachological adherence and increasing the identification of symptoms of several psychiatric and psychological disorders (Gastaldon et al., 2019). Colom and Vieta (2006) introduced a group psychoeducational program for patients with bipolar disorder. The target of this psychoeducational program consisted in illness awareness, adherence to pharmacological treatment, identification of prodromal symptoms and recurrences, and lifestyle regularity. Adding psychoeducation to other forms of psychiatric treatment has the advantage of helping patients and family members to understand their creative and positive role in the treatment and enhancing their ability to deal with daily stress (Pitschel-Walz et al., 2001). Psychoeducation is an adjunctive approach that has the potential to reduce the incidence of relapse, as well as rehospitalization rates and the mental health costs involved in relapsing (Shah et al., 2014; Joas et al., 2019).

While psychoeducational interventions are used for addressing adherence problems in psychiatric patients and are used in the context of suicide prevention (Battle, 1984; Jones et al., 2018), only a few studies have investigated the effects of psychoeducation interventions for suicide survivors. Wittouck et al. (2011) examined the effects of a cognitive-behavioral therapy-based psychoeducational approach on several psychological symptoms in 83 suicide survivors. However, the results showed that there were no significant effects of the psychoeducational intervention on depressive symptoms, complicated grief, and suicide risk factors. The authors suggested that the intervention may be useful as supportive counseling for suicide survivors. To better understand the pain of suicide survivors, Battle (1984) examined 36 survivors before and after group therapy, comparing them with 13 survivors who did not participate in the group and 31 patients in psychotherapy who were not bereaved. The authors focused on the differences in feelings experienced by the patients, noting how the predominant problem of the suicide survivors who participated in the group was their sense of guilt about the loved one’s death, while patients who did not participate in the group were also saddened by their loss but did not blame themselves. The authors found that the assumptions that frequently caused greater pain were: (1) those in which the survivor felt “I was passive while my beloved faced defeat and finally was beaten,” and (2) those that occurred when “the surviving parents, and others who knew the victim as a child, felt that the child rejected any identification that he/she may have had with them or that their child was not close to them in the first place.” More recently, Battuello and Milelli (2015), in a preliminary study of six suicide survivors who underwent group psychotherapy, found that, in the course of the therapy, patients learned to deal with their psychological pain better. Patients learned to deal with, including the grief and other psychological symptoms, learning how to begin life again after the dramatic loss from suicide.

Several mechanisms can explain the effects of psychoeducational interventions, including the group experience itself, the educational and informative sessions on symptoms and adequate treatments, some other generic features of the psychotherapeutic effect, or the combination of all three mechanisms. A group setting allows patients to verbalize experiences and emotions and acquire expertise and awareness about their symptoms and psychological suffering. The personal characteristics and clinical expertise of the group leader are important for enhancing the therapeutic effect of the group approach. A psychoeducational approach stresses a more “medical” view of psychiatric and psychological suffering, considering the biological, social, and relational aspects of each disorder. Several papers have suggested that group psychoeducation therapy promotes pharmachological adherence (Colom et al., 2003). Other relational, social, and non-pharmacological therapeutic aspects of the psychoeducational intervention are also fundamental in the effectiveness of this therapy (Motlova et al., 2017). Relational and social aspects include understanding and recognizing personal triggers and prodromal symptoms, lifestyle interventions, working on problem-solving and self-management techniques, and improving social support.

### A Personalized Psychoeducational Approach for Survivors

We developed a model of group psychoeducation starting from the psychoeducational program developed by Colom and Vieta (2006) for people with bipolar disorder. Even though the psychoeducational program has been validated in bipolar disorder, bipolar disorder is a different condition from suicide survival (Miklowitz and Scott, 2009). The main objectives of our program were to provide psychological support, decrease the abnormal reactions of family members, assist suicide survivors in recognizing their feelings of confusion, guilt, or anger, focus on their suicidal risk, and implement prevention strategies (Table 1).

**TABLE 1:** The 21 sessions of the group psychoeducation program for survivors.

1. Introduction: objectives and rules
2. Group presentation
3. Definition of suicide survivor
4. Sharing the pain caused by suicide
5. Psychological needs of survivors
6. Traumatic aftermath of suicide
7. Recurring thoughts
8. Stigma, shame, and isolation
9. Exploring unfinished issues in relationship with the deceased
10. Aiding in coping with divergent reactions among family members
11. Assessment of psychiatric symptoms
12. Understanding psychiatric symptoms
13. Risks associated with psychiatric symptoms
14. Talking about suicide
15. Assessment of suicide risk
16. Prevention of suicide risk
17. Stress-management techniques
18. Problem-solving techniques
19. Interpersonal relations and social support
20. Evaluation of the objectives
21. Final session

The group approach, compared to the individual approach, produces several benefits by creating an interpersonal space in which survivors tell of emotional, sensory, and mental experiences. This approach allows survivors to interact with each other following the rules of therapy and to express and share emotions, feelings, and expectations. In this framework, the group provides a personal experience, promoting the development of relationships between individuals who are in the same situation of deep psychological pain. According to Yalom and Leszcz (2005), some of the therapeutic factors involved in the effectiveness of group therapy involve the sense of awareness of experiences also lived by others, the sharing of new information, the increase of hope and altruism, and the increase in mastery of relationship techniques, imitative behavior, interpersonal learning, group cohesion, and psychological adaptation to the same traumatic experience. Before entering the group, survivors have experienced, due to the suicide death, an increase in stigma and social isolation, which, in turn, result in the development and worsening of their psychological pain, distress, and psychiatric symptoms. Through the sharing of information, the use of socializing techniques, self-disclosure, verbalization of emotions and feelings, acquiring adaptive coping mechanisms and restructuring behavioral techniques, the psychoeducational group approach creates a sense of cohesion that allows survivors to improve. Furthermore, in a group approach, the recognition by individuals who suffered the death of a loved person from the suicide of both similarities and differences helps, not only to diminish the sense of loneliness, but also to develop metacognitive skills.

Research has demonstrated that several aspects of the psychological pain experienced by suicide survivors differ from other types of traumatic losses (Jordan, 2001), including the clinical expectations of the therapist who is not used to working with suicide survivors. The competence to recognize the importance of pain (to be “present” with the pain) and to understand the psychological needs of suicide survivors is fundamental in approaching clients with suicide bereavement. The objectives of our psychoeducational therapy include primarily the integration of the loss into the survivor’s life. In this sense, the approach we use should be considered to be an accompaniment promoted by the therapist with the aim of allowing survivors to learn how to reinvest in life. Other features include carrying the loss, focusing on guilt and abandonment, and working on a positive therapeutic relationship to facilitate the healing process. Furthermore, psychoeducation helps suicide survivors, in particular, families, understand the nature of psychiatric distress and their role in contributing to the genesis of the psychological pain that led to suicide, allowing them to understand and internalize the death and acknowledge the devastating systemic impact of suicide on family systems.

**FIGURE 1:**
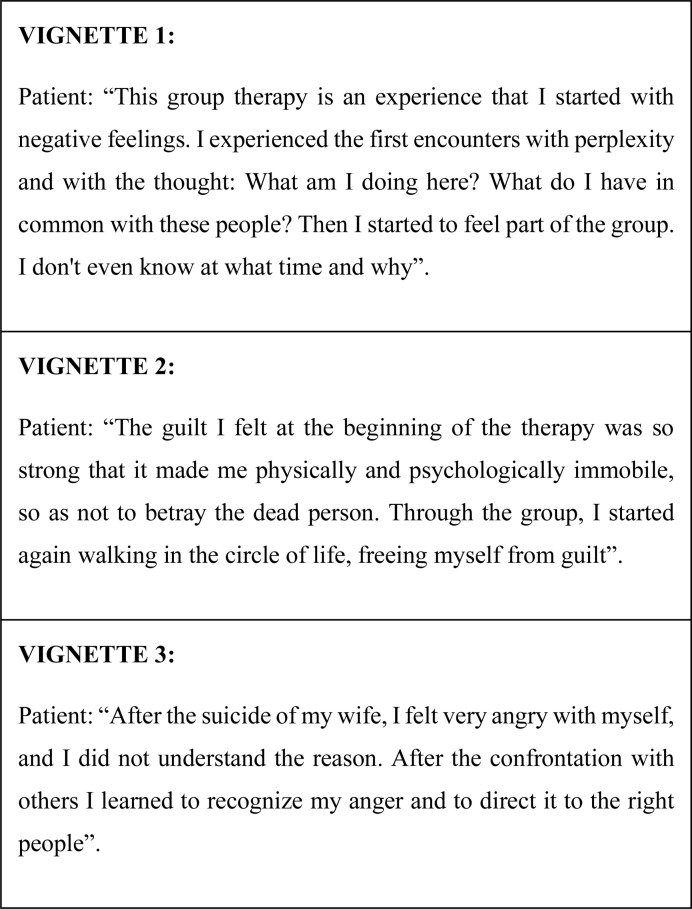
Examples of participants’ comments.

For suicide survivors, group psychoeducation can create a deep sense of awareness of experiences lived by others, and the social cohesion of the group, acting directly and indirectly on stigma and social isolation, helps suicide survivors to verbalize information and sharing their concerns in a framework of shared hope (Center for Substance Abuse Treatment, 1999). The effects experienced by each individual in the group and by the group itself are identified during the psychoeducational experience. It is necessary that the therapists are also aware that, sometimes, the group can limit the freedom of people by imposing an adjustment to a required collective functioning both in terms of emotions and thought imposed by other members of the group (Figure 1).

Our psychoeducational group was a homogeneous, finite-open group (Cerel et al., 2009). All the participants were survivors of suicide and, in the therapeutic program, new members can be accepted as additional resources into the group. The participants are usually referred to the Suicide Prevention Center in Rome, and the Director of the center decides on the acceptance of the survivors, keeping in mind who can benefit from a psychoeducation group therapy and also considering the composition of individuals who can benefit most from a group approach (the composition of psychoeducational group). Deciding, whether to include a person in a group, involves not only the relation between the therapist and that person but also between the other members of the group and that person. It is known that not everyone has the appropriate characteristics for joining a therapeutic group. Sometimes, the relational dynamics of a group can directly contribute to adverse outcomes for some patients, including worsening the psychological distress as a result of one’s group experience (Yalom and Leszcz, 2005).

A finite-open group offers a temporally defined experience for the group by programming its activity into a set number of educational sessions. In the first session of the psychoeducational therapy group, the therapeutic objectives of the group, and the roles of the therapist are clearly stated for the participants. The therapists in our psychoeducational program were two psychologists who work in the Suicide Prevention Center and who have dealt with suicide prevention for many years. The therapists lead the group, established its structure, and received weekly supervision from the Director of the Suicide Prevention Center. The two therapists in our program had to ensure that the psychological suffering of the survivors will be identified by the group, explored and understood by the group, and recognized as the object of group responsibility. The psychoeducational approach was composed of 21 sessions of 90 min, delivered weekly by the two therapists. The number of survivors ranged between 10 and 12 participants. Several areas were taken into account, including psychological suffering, emotional reaction to bereavement, guilt, stigma, any psychiatric symptoms such as depression, post-traumatic anxiety symptoms, possible risk of suicide, lifestyle regularity, interpersonal context, and social support. The topics of the sessions are summarized in Table 1. Each session begins with a 30-min talk in which the therapist presents the topic of the day, followed a period in which patients discuss the topic and share their experiences, resulting in a group discussion on feelings, emotion, and psychological distress.

The first session is an introductory session in which therapists introduce themselves and, together with the survivors, define the rules of therapy and the main objectives of the procedure. Sessions 2–9 are focused on evaluating and understand the mental state and the suffering experienced by the survivors (see Table 2). Therapists inform survivors about their condition and the emotional impact caused by the suicide of a loved one, working on feelings such as anger and fear, behaviors, and stigma. Session 10 focuses on the different reactions to suicide in the family and on the impact of suicide on family relationships. Sessions 11–13 are mainly focused on the assessment of information about several psychiatric symptoms that can develop after a suicide, including depressive symptoms, anxiety, and post-traumatic symptoms. The topic of sessions 14–16 is on suicide risk. The therapists assess and inform the survivors of the possibility of their own suicidal risk.

**TABLE 2:** Investigating the relationship between the suicide and the survivors (sessions 1–9). Main points.

Description of the relationship with the suicide, the degree of relationship, as well as the type of bond.
Dwell on the time that has elapsed since the suicide and on the events that have occurred during this period of time.
Investigate how many people are aware of the suicide and whether there are unrevealed events.
Promote the telling of what happened (who found the body, the method used).
Accept the spontaneous intuitions of the survivors regarding what happened.
Explore the day of the suicide (if the survivor had noticed something significant, if the suicide had tried to ask for help).
Have the group verbalize the emotional impact of the story that they have just heard.

Psychoeducational action is conveyed through relationships and the mediation of emotional-affective and body language, which fosters the emergence of images present within time and appropriate space in which they can be expressed and welcomed. The methodological approach supports non-verbal rehabilitation actions, working on the development of processes of personal re-working and self-awareness. As Shneidman proposed, the matrix of suicide lies in mental pain (psychache), a synthesis of painful emotions from which the person cannot escape. Suicide becomes, as Shneidman points out, not a movement toward death, but a movement away from the mental pain. In these sessions, therapists are able to focus on the assessment of psychological pain and suicide risk. The use of bodily experiences, inspired by the body-analytical model involving bioenergetic exercises, as an integral part of the psychoeducational group, enables participants to experience a particular form of learning and, in addition, provides a first level stimulus, activates processes that allow them to become aware of intrapsychic and relational dimensions, and facilitates the acquisition of new ways of thinking, feeling, and relating. Contact with the body represents a chance to give life to thoughts and give words to behavioral events. The involvement of the body allows putting the patient in contact with their own mental pain in order to recognize it, instead of denying it. The group offers a space for discussion of self-injurious behaviors and attempted suicide. This allows each individual to understand how their actions and impulses are perceived by others. Consideration of the perceptions of others enables each individual to gain an understanding that is more complex and realistic, including their effects on others and their own role in tackling current problems.

In sessions 17–19, the therapists address the important role of social support from family and friends by teaching general psychosocial skills using the relationships between the members of the group. In these sessions, skills training on how to effectively communicate with other people and understand social connections is offered to the group.

The psychoeducational program ends with sessions dedicated to outcome measures in different categories, including self-reports of distress, psychiatric symptoms, suicidal risk, and functioning and role appraisal. Furthermore, the survivors are assessed on how well they achieved the goals outlined by the therapist at the beginning of the group.

## Conclusion

Given the special thematic aspects of suicide bereavement, the unique pain of suicide survivors and the stigma that many survivors perceive in their social networks, the psychoeducational group approach we developed for them offers the opportunity to interact with other suicide survivors to resume the normal course of life and place the suicide of the significant other in a broader perspective. Therapists had to deal both with grief and monitoring such risk as well as proper assessment of psychiatric disorders. The psychoeducational program we propose for suicide survivors needs to be tested with different groups in different centers.

Psychoeducation educates survivors about the nature of suicide and the psychological pain associated with suicide, and endeavors to make sense of the suicide with the help of techniques including oral presentations, reading materials, and physical exercises. Finally, several sessions are focused—on social and family problems, often present in suicide survivors, helping them cope with their family and the social network. Limitations of the program include the need that participants should be cooperative with the therapists, the duration of the program which may last several months, and the need to have two trained therapists in the field of suicide. In managing our psychoeducational approach, considerable difficulty was found in those suicide survivors, who reacted to the suicide with positive emotions. In discussing the suicide of a difficult colleague, Dover (1994) noted that he felt happy after the individual died by suicide. In his discussion of this case, Lester (2005) noted that positive emotions might sometimes be present in the survivors of suicides, but survivors may feel that there is a stigma attached to revealing these positive emotions. For example, the wife and children of an alcoholic, who physically abused his family, may feel relief if the man dies by suicide. However, since the typical emotions expressed by survivors are grief and self-blame for not recognizing the suicide’s distress, the wife and children may feel that they would be judged harshly if they expressed their true feelings.

Suicidal individuals are not always easy to live with, and counselors helping survivors should bear in mind that relief and other positive emotions may be present as the primary emotion (Turchi et al., 2019). The counselor’s task in these situations is to make sure that the survivors do not feel guilt over such emotions. Lester (1995) also noted that the emotions present in those whose significant other choose assisted suicide might differ from the typical survivor, and counselors must be ready to assist those involved, both the suicide and the significant others, in the time preceding the suicide and afterward.

## Author Contributions

IB, DE, and DL drafted the manuscript. ER and SS collected the data and summarized results. MP designed the study. All authors contributed in drafting the final version of the manuscript.

## Conflict of Interest

The authors declare that the research was conducted in the absence of any commercial or financial relationships that could be construed as a potential conflict of interest.
